# Multi-Proxy Temperature Reconstruction from the West Qinling Mountains, China, for the Past 500 Years

**DOI:** 10.1371/journal.pone.0057638

**Published:** 2013-02-22

**Authors:** Fengmei Yang, Naiang Wang, Feng Shi, Fredrik Charpentier Ljungqvist, Shigong Wang, Zexin Fan, Junwei Lu

**Affiliations:** 1 Key Laboratory for Semi-Arid Climate Change of the Ministry of Education, College of Atmospheric Sciences, Lanzhou University, Lanzhou, China; 2 China Meteorological Administration Training Centre, Beijing, China; 3 Center for Climate Change and Hydrologic Cycle in Arid Region, College of Earth and Environmental Science, Lanzhou University, Lanzhou, China; 4 State Key Laboratory of Numerical Modelling for Atmospheric Sciences and Geophysical Fluid Dynamics, Institute of Atmospheric Physics, Chinese Academy of Sciences, Beijing, China; 5 Key Laboratory of Desert and Desertification, Cold and Arid Regions Environmental and Engineering Research Institute, Chinese Academy of Sciences, Lanzhou, China; 6 Department of History, Stockholm University, Stockholm, Sweden; 7 Key Laboratory of Tropical Forest Ecology, Xishuangbanna Tropical Botanical Garden, Chinese Academy of Sciences, Kunming, China; DOE Pacific Northwest National Laboratory, United States of America

## Abstract

A total of 290 tree-ring samples, collected from six sites in the West Qinling Mountains of China, were used to develop six new standard tree-ring chronologies. In addition, 73 proxy records were assembled in collaboration with Chinese and international scholars, from 27 publically available proxy records and 40 tree-ring chronologies that are not available in public datasets. These records were used to reconstruct annual mean temperature variability in the West Qinling Mountains over the past 500 years (AD 1500–1995), using a modified point-by-point regression (hybrid PPR) method. The results demonstrate that the hybrid PPR method successfully integrates the temperature signals from different types of proxies, and that the method preserves a high degree of low-frequency variability. The reconstruction shows greater temperature variability in the West Qinling Mountains than has been found in previous studies. Our temperature reconstruction for this region shows: 1) five distinct cold periods, at approximately AD 1520–1535, AD 1560–1575, AD 1610–1620, AD 1850–1875 and AD 1965–1985, and four warm periods, at approximately AD 1645–1660, AD 1705–1725, AD 1785–1795 and AD 1920–1945; 2) that in this region, the 20^th^ century was not the warmest period of the past 500 years; and 3) that a dominant and persistent oscillation of ca. 64 years is significantly identified in the 1640–1790 period.

## Introduction

Temperature-sensitive proxy data can be used as a primary basis for understanding temperature variations through time. Tree-ring datasets are particularly important natural proxy records used in climatological research, as they provide accurate dating, annual-scale resolution, and are available from locations distributed worldwide [1]. A major focus in dendroclimatology has been the reconstruction of temperatures at a single location using local tree-ring samples [2]–[15].

Despite the advantages of tree-ring records as temperature proxies, tree-ring chronologies often include less low-frequency information than do other proxies because of the so-called segment-length curse problem [16], which refers to the difficulty of preserving cyclic signals that are longer than the duration of the age of individual trees. Samples from individual trees rarely span time frames longer than a millennium, and are usually much shorter. The standardization process which is used to eliminate the effects of individual tree growth patterns, tends to weaken low-frequency climate signals [17]. Although dating errors of other proxy types are usually greater than those obtained from tree ring-data, other proxies are often more accurate in preserving low-frequency temperature signals [18]. Therefore, in this study, we employed a hybrid reconstruction method that considers the full spectrum of temperature variability, by separating the variability into high- and low-frequency bandwidths. Every proxy record contains both climate-related information and non-climate-related “noise”. In the proxy records of individual trees, the noise can be large and can even dominate the climate signal; however, when averaging a number of proxies, this noise is reduced (assuming that the noise is random). By assembling a large number of proxy records from the same region, we are more likely to extract a common climate-related signal, and the signal is likely to be less strongly affected by the noise inherent in individual proxies.

The correlations that exist between proxy data and instrumental data in Asia are complex [19]. For example, tree-ring width chronologies from the northeastern Tibetan Plateau (Dulan County), where elevation gradients are large, have been used to reconstruct precipitation [6], [20], temperature [3], [21], and the Palmer drought severity index (PDSI) [22]. Trees growing near the upper treeline typically respond to temperature, whereas those near the lower treeline respond to precipitation [23]. Furthermore, the complex monsoon system and the thermal forcing of the Tibetan Plateau may strongly influence Asian climate [24]. By using a large number of proxies to rebuild the response function through an ensemble reconstruction approach that averages a large number of ensemble members, the accuracy of the reconstructions may be improved [19].

We developed six new tree-ring chronologies from the West Qinling Mountains, and assembled 67 other proxy records in China. Using a modified point-by-point regression (hybrid-PPR) method, we reconstructed the annual mean temperatures over the past 500 years. Special attention was paid to the reconstruction method, as it differs slightly from those used in previous studies [19], [25].

## Materials and Methods

he West Qinling Mountains (33°–35°N, 102°–105°E) is situated near the boundary of the East Asia monsoon region, in the transition zone between the Tibetan Plateau and the Loess Plateau of China [26]. A multi-proxy approach is required to accurately reconstruct past climate variability in this region because of the complexity of the climate regime and the variability associated with different climate signals from this area. No specific permits were required for the described field studies. Permission to obtain tree-ring samples was granted by the Bailong River Forestry Bureau to the Lanzhou University research team headed by Dr. Fengmei Yang (first author). The study area belong to the Bailong River Forestry Bureaus This location is not privately owned or protected in any way, and the field studies did not involve endangered or protected species.

### 1. Instrumental data

**Figure 1:**
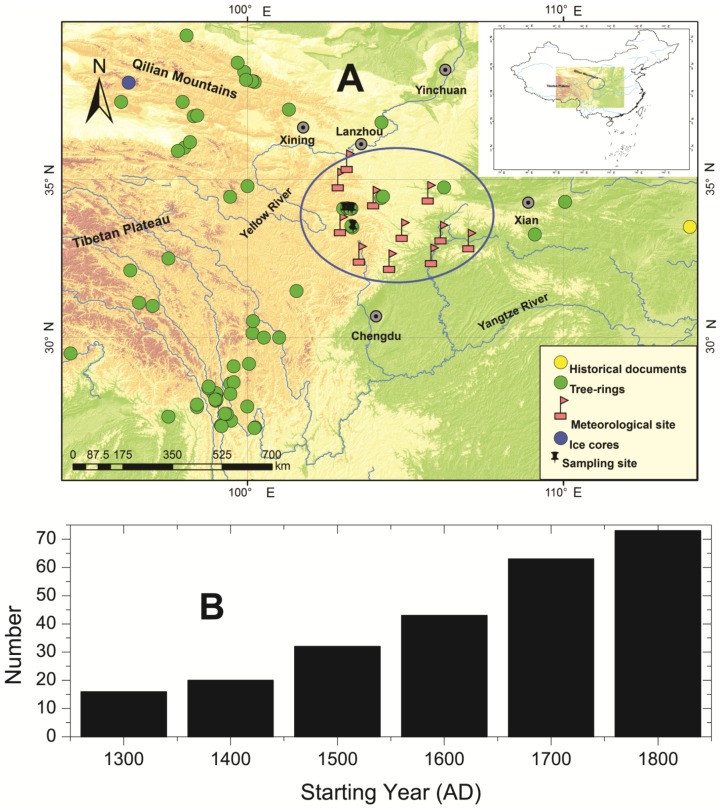
Characteristics of the proxy records. **A**) Map showing the study area and geographical locations of the 73 proxy records used in this study. **B**) The frequency per century of the start year of the proxy records.

We calibrated our temperature reconstructions using three different instrumental surface-air temperature datasets: the CRUTEM3v 5°×5° gridded instrumental surface-air temperature data set, which covers the period 1850–2006 (Climatic Research Unit, Norwich, UK; www.cru.uea.ac.uk/cru/data/temperature) [27]; the CRUTS3.1 0.5°×0.5° gridded instrumental surface-air temperature dataset, which covers the period 1901–2009 (Climatic Research Unit, Norwich, UK; http://badc.nerc.ac.uk/browse/badc/cru/data/cru_ts_3.10); and the average of the annual mean measured temperature for the period 1951–2006 obtained from 12 meteorological stations (Baoji, Guangyuan, Hanzhoung, Linxia, Lueyang, Heji, Minxian, Pingwu, Ruoergai, Songpan, Tianshui and Wudu) located in the West Qinling Mountains and the surrounding area (China Meteorological Data Sharing Service System, http://cdc.cma.gov.cn/) (see Figure 1). The quality of the meteorological data is strictly controlled, showing good uniformity and accuracy.

**Figure 2:**
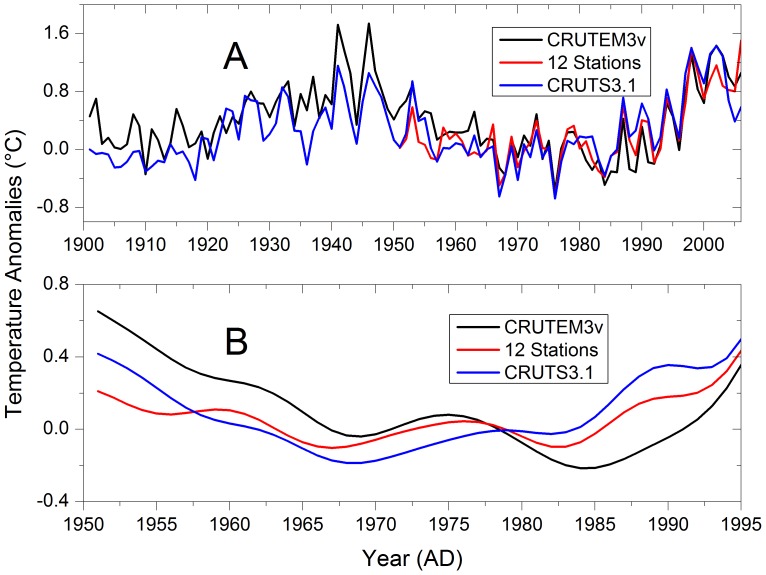
Comparison of three annual instrumental temperature series from the West Qinling Mountains, in China. **A**) The three annual instrumental temperature series (CRUTEM3v, CRUTS3.1, and the annual mean instrumental temperature anomalies, averaged from measurements at 12 local meteorological stations) for the period AD 1901–2006. **B**) The three instrumental series for the period AD 1951–1995, after low-pass filtering with a Butterworth IIR filter with a cut-off frequency of 0.1 cycles per year.

For the CRUTEM3v dataset we used a grid cell centred at 32.5°N and 102.5°E, and for the CRUTS3.1 dataset we used the four grid cells nearest to the West Qinling Mountains. We also used a grid cell centred at 33.75°N 103.75°E, as a correlation analysis between the CRUTS3.1 dataset and the averaged 12-meteorological-station dataset showed this grid cell to have the highest correlation coefficient for the period 1951–1995 (*r* = 0.85). In Figure 2A, the three instrumental temperature datasets seem similar, but the amplitude of the low-frequency variability differs greatly, as shown in Figure 2B. The amplitude of the temperature variability in the CRUTEM3v data after AD 1966 is clearly greater than is evident in the averaged data of the 12 meteorological stations. Similarly, the amplitude of the temperature variability in the CRUTS3.1 data after AD 1957 is slightly greater than that observed in the averaged data of the 12 meteorological stations. The variability between the different instrumental datasets is of importance, as the differences have a strong impact on the reconstruction results.

### 2. Temperature proxy data

The West Qinling Mountains are relatively free from human disturbance and the trees are mostly healthy, thus providing ideal conditions for obtaining for dendroclimatological records from tree-ring samples. During 2009 and 2010, we collected 290 tree-ring samples obtained as increment cores at six sites in the West Qinling Mountains, and from these data, we developed six new multi-centennial tree-ring width chronologies (see Figure 1). The ring widths in the cross-dated samples were measured and the initial dating (and associated errors) of the samples were cross-checked using the quality-control software COFECHA to check the cross-dating and overall quality of the tree-ring chronologies [28]. The ring-width measurement series were detrended by applying a negative exponential curve; some tree-ring width series which did not exhibit negative exponential growth trends were excluded when the chronologies were developed. The chronologies were calculated from the bi-weighted robust means of the raw measurement data using the dendrochronology program library in R (dplR) [29]. The standard version of the six chronologies in which the expressed population signal (EPS) exceeds a threshold value of 0.85 [30] was assessed for coherence using correlation analysis.

In addition to the six new tree-ring chronologies, we collected 67 other multi-proxy records from the surrounding area to use in the reconstruction (see Figure 1 and Table 1), including 21 published tree-ring chronologies were archived in National Climatic Data Center (NCDC) Paleoclimatology (http://www.ncdc.noaa.gov/paleo/paleo.html), 13 of which were shared by Asia 2K regional group, as part of the PAst Global changES (PAGES) 2K Network, and recently were archived in the International Tree-Ring Data Bank (ITRDB; http://www.ncdc.noaa.gov/paleo/treering.html). Two tree-ring chronologies were derived from the Chinese Tree Ring Proxy Dataset (CTRPD; http://cdc.cma.gov.cn/home.do). Moreover, two tree-ring chronologies were obtained from the Chinese Tree Ring Data Center (CTRDC; http://ctrdb.ibcas.ac.cn/index.asp). The 28 chronologies are described in 20 peer-reviewed articles [3], [6], [31]–[48]. The remaining 14 unpublished tree-ring chronologies came from the co-authors and were firstly used for the multi-proxy temperature reconstruction.

**Table 1:** List of the 73 proxy series used in this study.

No.	Site/ID	Lat.	Lon.	Type	Resolution	Start year	End year	Source/References
**1**	chin001a	37.0	100.0	Tree rings	Annual	1163	1986	ITRDB extraction
**2**	chin003a	38.1	100.2	Tree rings	Annual	1414	1986	ITRDB extraction
**3**	chin004a	34.3	110.1	Tree rings	Annual	1542	1989	ITRDB extraction
**4**	chin004ea	34.3	110.1	Tree rings	Annual	1562	1988	ITRDB extraction
**5**	chin004la	34.3	110.1	Tree rings	Annual	1561	1988	ITRDB extraction
**6**	chin004xa	34.3	110.1	Tree rings	Annual	1561	1988	ITRDB extraction
**7**	chin005	37.0	98.3	Tree rings	Annual	840	1993	ITRDB extraction
**8**	chin006	36.0	98.0	Tree rings	Annual	159	1993	ITRDB extraction
**9**	chin016	31.5	101.6	Tree rings	Annual	1575	2007	Asia2K, ITRDB extraction
**10**	chin017	28.5	99.5	Tree rings	Annual	1452	2007	Asia2K, ITRDB extraction
**11**	chin018	29.2	100.1	Tree rings	Annual	1540	2006	Asia2K, ITRDB extraction
**12**	chin019	29.1	99.6	Tree rings	Annual	1509	2006	Asia2K, ITRDB extraction
**13**	chin020	30.1	100.2	Tree rings	Annual	1306	2007	Asia2K, ITRDB extraction
**14**	chin021	28.6	99.6	Tree rings	Annual	1380	2007	Asia2K, ITRDB extraction
**15**	chin022	30.5	100.2	Tree rings	Annual	1663	2007	Asia2K, ITRDB extraction
**16**	chin023	30.0	100.5	Tree rings	Annual	1715	2007	Asia2K, ITRDB extraction
**17**	chin024	28.2	99.0	Tree rings	Annual	1628	2007	Asia2K, ITRDB extraction
**18**	chin025	27.2	99.2	Tree rings	Annual	1483	2007	Asia2K, ITRDB extraction
**19**	chin026	27.4	99.5	Tree rings	Annual	1516	2007	Asia2K, ITRDB extraction
**20**	chin027	27.2	99.2	Tree rings	Annual	1348	2007	Asia2K, ITRDB extraction
**21**	chin028	27.2	99.2	Tree rings	Annual	1348	2007	Asia2K, ITRDB extraction
**22**	Fukang A	37.2	101.3	Tree rings	Annual	1665	1994	CTRPD
**23**	Binggou	38.1	100.2	Tree rings	Annual	1259	1994	CTRPD
**24**	Delingha	37.5	98.0	Tree rings	Annual	1010	2001	CTRDC
**25**	Wulan	37.0	98.4	Tree rings	Annual	20	2000	CTRDC
**26**	Zhenan	33.3	109.1	Tree rings	Annual	1666	1993	[44]
**27**	Chuanxi	30.0	101.0	Tree rings	Annual	1650	1995	[45]
**28**	Jingyuan	36.8	104.2	Tree rings	Annual	1698	2010	[46]
**29**	Linzhi	29.5	94.4	Tree rings	Annual	1624	1998	[47]
**30**	Maqen	34.5	99.5	Tree rings	Annual	1173	2001	[48]
**31**	Qilian-hug	38.1	100.2	Tree rings	Annual	450	2008	[31]
**32**	Qilian-neg	38.1	100.2	Tree rings	Annual	632	2008	[31]
**33**	Qilian-rcs	38.1	100.2	Tree rings	Annual	575	2008	[31]
**34**	Dulan	35.9	97.8	Tree rings	Annual	1	2000	[6]
**35**	Qamdo	31.0	97.0	Tree rings	Annual	1000	1996	[32]
**36**	Gaoligong	27.8	98.4	Tree rings	Annual	1585	1996	[34]
**37**	Delingha	37.5	96.0	Tree rings	Annual	1450	2006	[33]
**38**	Sidalong	38.4	99.9	Tree rings	Annual	1450	2006	[33]
**39**	Haiyagou	38.7	99.7	Tree rings	Annual	1450	2006	[33]
**40**	Qifeng	39.6	98.1	Tree rings	Annual	1450	2001	[33]
**41**	Anemaqin	34.8	100.0	Tree rings	Annual	1450	1996	[33]
**42**	Zhamashike	38.2	100.0	Tree rings	Annual	1450	1996	[33]
**43**	MXD Age banded regional lowf added	32.5	97.5	Tree rings	Annual	1406	1996	[35]
**44**	MXD Age banded regional lowf added	27.5	97.5	Tree rings	Annual	1453	1996	[35]
**45**	Dulan	36.2	98.2	Tree rings	Annual	1000	2000	[3]
**46**	Qamdo	31.1	96.6	Tree rings	Annual	1000	2010	[36]
**47**	Zhongdian	28.2	99.5	Tree rings	Annual	1475	2003	[37]
**48**	Deqen	28.0	99.0	Tree rings	Annual	1750	2006	[38]
**49**	Nangchen	32.1	96.3	Tree rings	Decadal	1624	2002	[39]
**50**	Xiaolong	34.8	106.2	Tree rings	Annual	1630	2009	[40]
**51**	Central east China	33.5	114.0	Document	Decadal	1000	1991	[41]
**52**	Dunde	38.1	96.2	Ice cores	Decadal	1607	1983	[42]
**53**	Dunde	38.1	96.2	Ice cores	Decadal	1005	1982	[43]
**54**	Chonger	34.1	103.0	Tree rings	Annual	1714	2010	This study*
**55**	Daban	33.5	103.3	Tree rings	Annual	1605	2010	This study*
**56**	Wuzi	34.1	103.2	Tree rings	Annual	1587	2010	This study*
**57**	Gaozhai	33.5	103.3	Tree rings	Annual	1679	2010	This study*
**58**	Yarika	34.1	103.3	Tree rings	Annual	1624	2010	This study*
**59**	Yazha	34.1	103.3	Tree rings	Annual	1715	2010	This study*
**60**	Longfeng	34.4	104.2	Tree rings	Annual	1788	2004	This study
**61**	Zhangxian	34.5	104.3	Tree rings	Annual	1696	2005	This study
**62**	4YL_A_r	27.2	100.2	Tree rings	Annual	1784	2005	This study
**63**	5BT_P_r	27.8	100.0	Tree rings	Annual	1651	2007	This study
**64**	6HP1_P_r	28.3	98.9	Tree rings	Annual	1724	2005	This study
**65**	7HP2_P_r	28.2	99.0	Tree rings	Annual	1738	2005	This study
**66**	8YB_P_r	28.4	98.8	Tree rings	Annual	1740	2003	This study
**67**	9YL_P_r	27.1	100.2	Tree rings	Annual	1658	2006	This study
**68**	10GK_P_r	27.6	99.4	Tree rings	Annual	1518	2005	This study
**69**	11YC_T_r	27.6	99.3	Tree rings	Annual	1658	2005	This study
**70**	12YM_A_r	28.0	99.0	Tree rings	Annual	1661	2005	This study
**71**	13YE_T_r	28.0	99.0	Tree rings	Annual	1642	2005	This study
**72**	14MY_P_r	28.5	98.8	Tree rings	Annual	1770	2005	This study
**73**	22DL_T_r	27.9	98.4	Tree rings	Annual	1612	2005	This study

* : six new chronologies; ITRDB extraction: the tree ring data are reprocessed from the International Tree-Ring Data Bank (ITRDB), IGBP PAGES/World Data Center for Paleoclimatology, NOAA/NGDC Paleoclimatology Program, Boulder, Colorado (http://www.ncdc.noaa.gov/paleo/treering.html).

All records were required to have decadal (or higher) temporal resolution. With the exception of some of the tree-ring chronologies, all of the proxies in this study have been previously used to reconstruct temperature variability records and all have been validated as temperature proxies [41]–[43]. We used only tree-ring records showing a strong correlation with the annual mean temperature of the 12 stations during the period AD 1951–1995, at a confidence level of 90% or above. All tree-ring chronologies were required to span the interval AD 1800–1980. Proxies with non-annual temporal resolutions were linearly interpolated to an annual resolution; this interpolation introduced a strong bias by adding red noise to the high-frequency components of the spectrum, thus reducing the verification skill because of the higher autocorrelation [49], [50]. The non-annually resolved proxy data were therefore used only to reconstruct low-frequency temperature variability.

### 3. Reconstruction methods

The temperature reconstruction method used in this study was a modified PPR method. The PPR is a straightforward principal component regression that has been successfully used to produce high-quality reconstructions of droughts in North America [51], and for generating the North American Drought Atlas [52] and the Monsoon Asia Drought Atlas [19]. The PPR is based on the principle that only proxy records close to the location being reconstructed are good predictors for that location; this premise is particularly important for places with a spatially heterogeneous climate, such as China. The traditional PPR approach uses two variables: search radius and screening probability. The search radius defines the maximum tolerable distance between potential proxy records and the location of the reconstruction. The 450 km search radius was found to be the optimal search radius [53]. The four search radii for the Palmer Drought Severity Index (PDSI) in Asia, 500 km, 1000 km, 2000 km and 3000 km were chosen in that study [19]. The reason for this is the irregular distribution and relatively high noise level of many tree-ring chronologies in China. In this work, search radii of 100, 300, 500 and 1000 km were used for the temperature reconstructions, as smaller search radii are likely to yield a more accurate representation of the local climate information.

The screening probability defines the correlation threshold for including proxy series in the temperature reconstruction. First, all proxies were screened against instrumental data and only those with correlations at or above a 90% confidence level were retained. Because of the high level of noise in much of the proxy data from China, the same weighted model was applied to the records in this study, as was implemented in the creation of the Monsoon Asia Drought Atlas [19].

Our approach differs from the traditional PPR approach by incorporating a “hybrid” procedure that separately calibrates information in “inter-decadal” and “inter-annual” bandwidths; inter-decadal bandwidths are those with periods longer than 10 years (including proxies with annual or decadal resolution) and inter-annual bandwidths are those with periods shorter than or equal to 10 years (including those with only annually resolved proxies). This process is derived from the hybrid frequency-domain calibration [50]. The final annual temperature reconstructions were assembled using the two frequency results. Our approach further differs from the traditional PPR in that we used the regularized errors-in-variables (EIV) method to allow for the assimilation of non-tree-ring data, instead of using the traditional principal component regression method. The steps are described in more detail below.

#### **3.1 Infilling the proxy data**

We used a cubic spline to interpolate proxy records with non-annual- to annual-scale resolutions. Proxies that did not extend to AD 1996 were extrapolated to AD 1996 using the regularized expectation maximization (RegEM) algorithm, based on their mutual covariance with the other available proxies over the 1800–1996 period [54]. In this study, 18 tree-ring chronologies, two ice-core records (No. 52 and 53 in Table 1) and a Chinese documentary record (No. 51 in Table 1) were extrapolated. Finally, to avoid biases due to different temporal resolutions and interpolation procedures, all records were filtered to retain frequencies of f<0.1 cycles per year in the reconstruction.

#### **3.2 Screening of proxy data**

The screening probability in original PPR frame is a method for selecting proxy records for use in the temperature reconstructions at a particular grid point, based on a threshold defined by a certain significance level for correlation [53]. Firstly, every proxy record was required to show a strong correlation (>90% confidence level; *n* = 45) with the annual temperature of the 12 meteorological stations for the period AD 1951–1995. Because of the high level of noise in the tree-ring chronologies, the same weighted model with the same coefficients was applied in this study as was used in the creation of the Monsoon Asia Drought Atlas [19]. This is expressed as
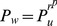
(1)


where 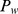 is the weighted proxy data, 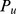 is the original un-weighted proxy data in standard normal deviate form, *r* is the correlation between the tree-ring chronologies and the instrumental temperature data over the calibration period 1951–1995, and *p* is some power, 0, 0.5, 0.67, 1, 1.5, 2, used to assign different weights to each proxy dataset, following the ensemble PPR method [19].

#### **3.3 Splitting of proxy and instrumental data**

Following the procedure described in the paper [55], the screened proxy and instrumental data were separated into high- and low-frequency bandwidths using a Butterworth IIR filter with a cut-off frequency of 0.1 cycle per year. The screened proxy records were used to reconstruct temperature in separate high- and low-frequency bandwidths. This frequency split makes it possible to assimilate proxy data series with different temporal resolutions.

#### **3.4 Regressing of proxy data**

The regularized EIV temperature reconstruction method [55] was used to reconstruct past temperature variability in the two frequency bandwidths, instead of the more traditional principal component regression method. These techniques are described in detail elsewhere [49], [54], [56]–[58]. We acknowledge the considerable debate in the literature about the validity of the RegEM/EIV method; however, the method has been utilized in numerous previous studies for reconstructing temperatures on local to global scales [49], [55], [58]–[62], and it is also used to reconstruct continental-scale temperature variability by the PAGES 2K network. We considere it to be a feasible method for rebuilding the transfer function. The code for the RegEM/EIV method is availible at http://www.meteo.psu.edu/holocene/public_html/supplements/Multiproxy

Means07/code/coderecon/. When applying small values of the search radius, the approach used here shares the reconstruction principles that form the basis of the PPR method [53]. Based on the EIV method, the regularized expectation maximization (RegEM) algorithm [49], [58] was used to determine a matrix of regression coefficients 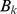 at grid point 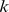 and model errors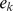, as
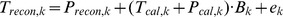
(2)


where 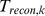 is a row vector giving the predicted temperature at grid point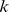, 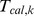 is a row vector giving the instrumental temperature during the calibration period, 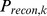 is a matrix of the proxy data (as the predictors), 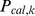 is a matrix of proxy data during the calibration period [53]; this equation is revised from this paper [49]. As a further safeguard against potentially non-robust results, a minimum of seven predictors is required in implementing the EIV procedure, according to the process described in this paper [55]. This criterion regarding the number of minimum predictors is an empirical constant, which is a compromise between the number of available predictors and the requirement for the model stability. For this reconstruction, the minimum number of predictors is greater than 20.

#### **3.5 Validation of reconstruction**

The accuracy and skill of the grid-point reconstructions were assessed using a split-period approach [19], [55]. Reconstruction models were validated for the period AD 1921–1950, and the fit of the reconstructions was measured by the square of the Pearson product-moment correlation coefficient (*r* ^2^), the reduction of error (RE), and the coefficient of efficiency (CE) [19]. The uncertainty of the reconstruction is expressed as in the paper [55], the code for this formula is available at Prof. Mann’s website (www.meteo.psu.edu/mann/supplements/MultiproxyMeans07/code/codeveri/

calc_error.m), and is expressed by
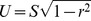
(3)


where U is the uncertainty, S is the standard deviation of the instrumental data during the calibration period, and *r* ^2^ is the squared correlation coefficient, calculated from the data for the verification period.

A wavelet analysis was used to detect cycles in our reconstruction results. The source code for the wavelet analysis is available at this website (http://paos.colorado.edu/research/wavelets/software.html) [63]. The mother function was set as the Morlet wavelet, which gives a high resolution for the periodicity and includes a complex exponential function modulated by a Gaussian wavelet [63]. A simple red-noise model representing an autoregressive linear Markov process [64] was chosen as an appropriate background spectrum for determining the 95% confidence levels of the global wavelet power spectrum. The autocorrelation parameter of red noise background is 0.72.

## Results and Discussion

Reconstruction accuracy as measured by *r* ^2^, RE and CE are shown in Table 2. We used four search radii (100, 300, 500 and 1000 km), and six weighted cases (powers of 0, 0.5, 0.67, 1, 1.5, and 2), giving 24 ensemble members. Our ensemble includes some members for which RE and CE exceed 0, as these values indicate that these parameters are of value [19]. Estimates of the uncertainty are included in Table 2, as based on equation 3. The CE values are mostly negative, indicating that the results are not well verified. The main reasons for this are the very short duration of the instrumental temperature data in the study area and the restricted ability of proxy data to extract the climate signals; thus, temperature is only one factor affecting tree-ring growth patterns in this region [65].

**Table 2:** Statistical validation of the reconstruction, skill assessments and uncertainty estimates.

		CRUTem3v				CRUTS3.1				Station			
Rad.	Wei.	RE	CE	r^2^	Un.	RE	CE	r^2^	Un.	RE	CE	r^2^	Un.
**100**	0	0.05	−0.74	0.04	0.33	0.40	0.31	0.37	0.26	0.25	0.14	0.25	0.29
**100**	0.5	0.26	−0.36	0.12	0.32	0.01	−0.14	0.16	0.30	−0.17	−0.35	0.05	0.32
**100**	0.67	−0.35	−1.47	0.00	0.34	−0.60	−0.84	0.01	0.33	−0.35	−0.55	0.06	0.32
**100**	1	−0.83	−2.35	0.01	0.33	−1.04	−1.35	0.05	0.32	−0.65	−0.90	0.03	0.32
**100**	1.5	−0.49	−1.72	0.06	0.33	−0.79	−1.06	0.02	0.33	−0.64	−0.89	0.01	0.33
**100**	2	0.15	−0.56	0.00	0.34	−0.23	−0.41	0.01	0.33	−0.21	−0.39	0.00	0.33
**300**	0	0.49	0.07	0.40	0.26	0.08	−0.06	0.32	0.27	0.05	−0.09	0.23	0.29
**300**	0.5	0.55	0.17	0.33	0.28	−0.21	−0.39	0.21	0.29	0.06	−0.08	0.15	0.30
**300**	0.67	0.28	−0.31	0.26	0.29	−0.65	−0.90	0.36	0.26	0.08	−0.06	0.26	0.28
**300**	1	−0.90	−2.47	0.05	0.33	−0.76	−1.03	0.13	0.31	−0.64	−0.88	0.06	0.32
**300**	1.5	−0.43	−1.61	0.01	0.34	−1.30	−1.65	0.01	0.33	−0.87	−1.15	0.00	0.33
**300**	2	−0.22	−1.23	0.00	0.34	−0.65	−0.90	0.02	0.33	−0.49	−0.72	0.01	0.33
**300**	0	0.17	−0.51	0.00	0.34	0.08	−0.05	0.11	0.31	0.03	−0.12	0.08	0.32
**300**	0.5	0.26	−0.35	0.02	0.33	0.17	0.05	0.25	0.29	−0.13	−0.30	0.08	0.32
**300**	0.67	0.11	−0.64	0.02	0.33	−0.19	−0.36	0.23	0.29	−0.04	−0.20	0.12	0.31
**300**	1	−0.27	−1.32	0.01	0.33	−0.11	−0.28	0.15	0.30	−0.27	−0.46	0.06	0.32
**300**	1.5	−0.66	−2.04	0.01	0.34	−1.32	−1.67	0.00	0.33	−0.72	−0.98	0.00	0.33
**300**	2	−0.22	−1.24	0.01	0.34	−0.61	−0.86	0.03	0.32	−0.43	−0.65	0.02	0.33
**1000**	0	0.25	−0.37	0.19	0.30	−0.92	−1.21	0.04	0.32	−0.20	−0.38	0.04	0.32
**1000**	0.5	0.06	−0.72	0.05	0.33	−0.81	−1.08	0.00	0.33	0.11	−0.03	0.05	0.32
**1000**	0.67	0.01	−0.81	0.00	0.34	−0.14	−0.31	0.06	0.32	0.16	0.03	0.10	0.31
**1000**	1	0.40	−0.09	0.24	0.29	0.17	0.05	0.32	0.27	0.17	0.05	0.20	0.29
**1000**	1.5	0.20	−0.46	0.00	0.34	0.04	−0.10	0.16	0.30	0.10	−0.03	0.11	0.31
**1000**	2	−0.06	−0.94	0.00	0.34	−0.52	−0.75	0.00	0.33	−0.43	−0.65	0.00	0.33
	Total	0.52	0.12	0.37	0.27	0.25	0.14	0.31	0.27	0.19	0.07	0.18	0.30

The 24 ensemble members include combinations of four search radii (100 km, 300 km, 500 km and 1000 km) and six weighted cases (using powers of 0, 0.5, 0.67, 1, 1.5 and 2 for weighting).

Rad. = search radii (km); Wei. = Weighted cases. Radi; Un. = uncertainty estimates; Total: we ensembled some parts of the 24 members in which RE and CE exceed 0, as these values of RE and CE exceeding 0 indicate that the reconstruction is reliable.

**Figure 3:**
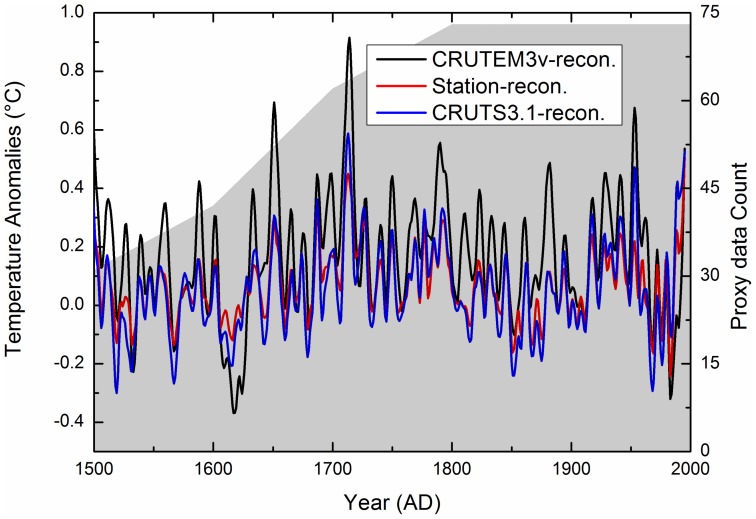
Reconstructed annual mean temperature anomalies (with reference to AD 1961–1990) for the past 500 years in the West Qinling Mountains, calibrated against three different instrumental datasets. The series were low-pass filtered by a Butterworth IIR filter with a cut-off frequency of 0.1 cycles per year.

Figure 3 shows reconstructed temperature anomalies (with respect to AD 1961–1990) for the past 500 years in the West Qinling Mountains in China, calibrated against three different instrumental datasets. The three results show similar trends. Cold excursions relative to from the underlying trend are present during five periods, at approximately AD 1520–1535, AD 1560–1575, AD 1610–1620, AD 1850–1875, and AD 1965–1985. Warm excursions are present during four periods, at approximately, AD 1645–1660, AD 1705–1725, AD 1785–1795, and AD 1920–1945. The last warm period (AD 1920–1945) is likely to be the regional expression of 20^th^ first century global climate warming, the similar example that the Arctic air temperature warming during the AD 1910–1940 period [66].

Our reconstructions show that the 20^th^ century was not warmer than other periods in the past 500 years in this region of China. The phases of the three reconstructions are mostly consistent, but the reconstruction that was calibrated against the CRUTEM3v dataset shows larger amplitudes than do the other two reconstructions. It is important to note, however, that the choice of instrumental data has a significant effect on the amplitude of the reconstructed temperature variations during the warm periods in the 18^th^ century. For example, for the CRUTEM3v dataset, the instrumental temperature (filtered using a Butterworth IIR filter with a cut-off frequency of 0.1 cycle per year) exhibits a distinctly and larger amplitude than other datasets (Figure 2B). When compared with the long-term means, the 1961–1990 reference period was relatively cold in the West Qinling Mountains, in contrast to most other regions in the Northern Hemisphere. Consequently, the reconstructed temperatures are above the 1961–1990 means for most of the past 500 years, which is the reverse of the situation observed in most other regions, including China as a whole.

**Figure 4:**
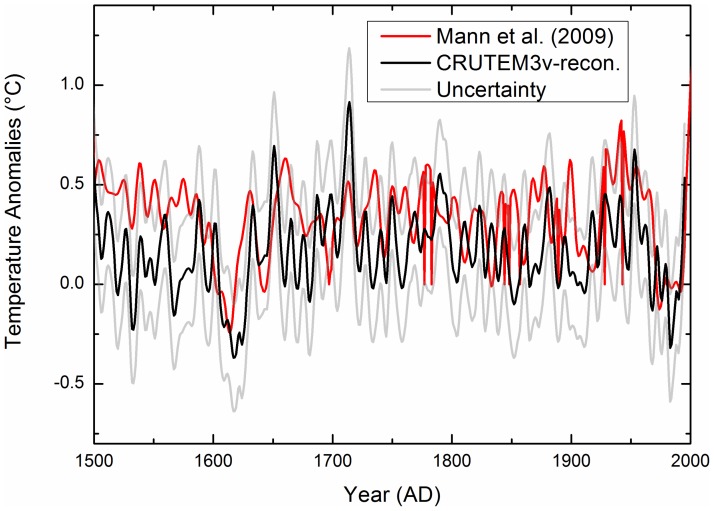
Comparison between our reconstruction and the other result [**67**] (using the CRUTEM3v dataset) for the last 500 years. The gray lines represent the uncertainties in the reconstruction.

Figure 4 compares of our reconstruction with the other results [67]; both studies used the same instrumental data and CRUTEM3v for the past 500 years. Grey lines show the uncertainties. Two reconstructions are presented, using the different proxy datasets. There are only several proxy records in the study area used in the other reconstruction [67]; however, we used 73 proxy records in this study. The reconstructions show two cold periods at ca. AD 1610–1620 and ca. AD 1965–1985, and two strong warming periods at ca. AD 1645–1660 and ca. AD 1920–1945. Overall, the timing of some of the transition periods from warm to cold conditions in our results coincides with the other results, but the timings of some transition periods do not match. The amplitudes of our reconstruction are larger than those [67] for the field temperature reconstruction of corresponding geographical regions (see Figure 4). The differences might be attributed to different methodologies and different proxy data. The PPR method has the advantage, at least in theory, of preserving local signals more accurately than do methods based on empirical orthogonal function regression [67]. In addition, in this study, we have used more local proxy data, which can preserve more distinct local climate variability, than the other result [67]. The use of a great number of local and high-quality proxy records will more realistically reflect the local climate variability in a given area.

**Figure 5:**
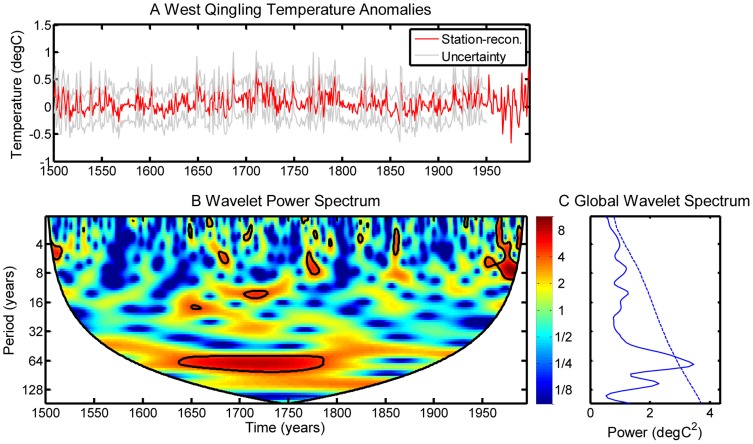
Wavelet analysis of our reconstruction. Top panel: surface-air temperature series during the period AD 1500–1995; Bottom panel: time-frequency values for the wavelet power. The bottom left panel shows the global spectrum of the wavelet power averaged over time. Dashed lines represent the significance level of the global spectrum, and are referenced to the power of the red noise level at the 95% confidence interval.

A wavelet analysis was used to detect cycles in the reconstructions over the full reconstruction period AD 1500–1995. The results (Figure 5) indicate that a dominant and persistent oscillation of ca. 64 years is present across almost the entire period, and is especially noticeable during ca. AD 1640–1790. This dominant timescale of climatic variability, which has been found in instrumental records [68], other proxy data [69] and simulation results [70], may originate from an internal variability of the ocean-atmosphere system, e.g. the Atlantic multi-decadal oscillation [71].

**Figure 6:**
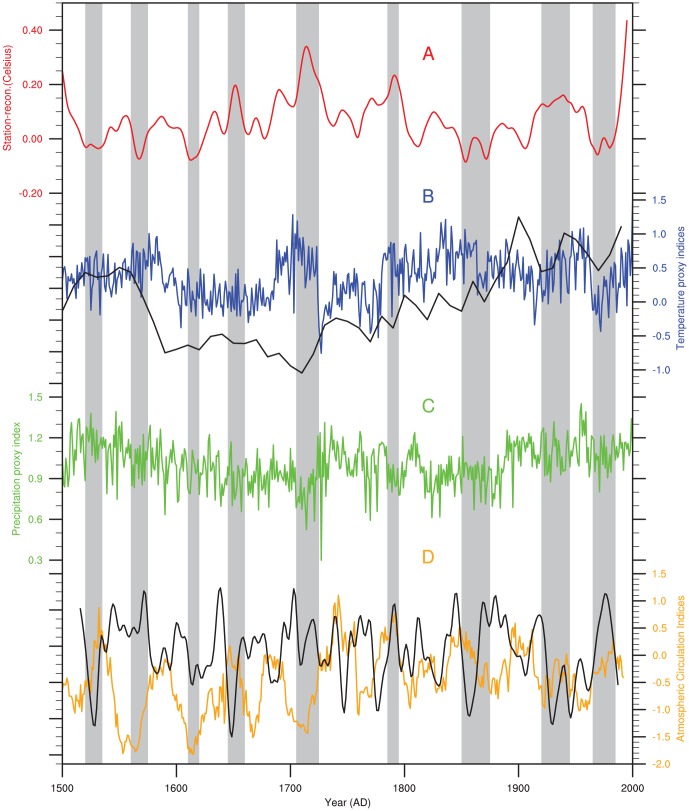
Comparisons among our temperature reconstruction and other reconstructions of temperature, precipitation, the El Niño-Southern Oscillation and the Pacific Decadal Oscillation. **A**) Station- reconstructed temperature in the West Qinling Mountains. **B**) Reconstructed temperature (blue line) in the Qamdo region of the Tibetan Plateau (blue line), calculated using the tree-ring regional curve standardization (RCS) chronology [36], and the reconstructed temperature (black line), using the tree ring chronology from the middle Qilian Mountains [5], [72]. **C**) Reconstructed precipitation and temperature (green line), calculated using the tree-ring chronology from Zhamashike in the Qilian Mountains [33]. **D**) Reconstructed ENSO (orange line), calculated using tree-ring chronology [73], and the reconstructed PDO (black line), calculated from tree-ring chronologies [74], filtered to retain frequencies above a cut-off frequency of 0.1 cycle per year.

Figure 6 compares between our temperature reconstruction with other reconstructions of temperature [5], [36], [72], precipitation [33], the El Niño-Southern Oscillation (ENSO) [73], and the Pacific Decadal Oscillation (PDO) [74]. The temperatures reconstructed for Sunan County are representative of the temperature variability for the central Qilian Mountains [5], [72]. The Qamdo temperature reconstruction is representative of the temperature variability for the southeast area of the Tibetan Plateau [36]. The precipitation reconstructed for Zhamashike correlates strongly with precipitation in other regions of the Qilian Mountains; this correlation is, significant at the 95% confidence level, indicating that this record is likely to represent a response to a common precipitation signal [33], and therefore used to represent precipitation variability in the Qilian Mountains. The ENSO variance reconstruction, which denotes the Niño3 region, is based on the North American Drought Atlas [73] and is derived from the tree-ring datasets in Northern America. The PDO index was developed from 17 Asian tree-ring chronologies [74], filtered to retain frequencies of *f* <0.1 cycle per year.

The dominant 1705–1725 warm period (Figure 6) coincides with a cold and dry period in the central Qilian Mountains and a warm period in the southeastern Tibetan Plateau and is related to lowered ENSO variance and raised PDO values. This period coincides with a weakened Asian summer monsoon [40], which is the main source of precipitation in the study area. Moreover, the temperatures during this period are greater than or equal to those in the 20^th^ century. These warm periods were evident in the south-eastern Tibetan Plateau [36]. The other three warm periods could be associated with dry periods in the Qilian Mountains and a strengthened Asian summer monsoon. This indicates that the Asian monsoonal dynamics may be a major factor affecting climate variability in the study area, but it is not the only factor. The last warm period occurred during the period AD 1920–1945. A widespread drought event occurred in the 1920s and 1930s in northern China [75], [76]; this can be observed in our reconstruction, although the duration of the event may have been longer in the study area, possibly representing divergences between local and regional environmental trends.

According to our reconstructions, the first three cold periods occurred at approximately AD 1520–1535, AD 1560–1575, and AD 1610–1620, all of which all belong to period of the Little Ice Age. The last cold period AD 1610–1620 can also be identified in the central Qilian Mountains and on the Tibetan Plateau, but the other cold periods can not be found (see the Figure 6). These patterns indicate that temperatures exhibited different phases and amplitudes in different areas during the Little Ice Age. The precipitation in the central Qilian Mountains was high during AD 1520–1535, but dry conditions prevailed during the next two cold periods. This indicates that warm periods and wet periods are not homogeneous in this area.

Temperatures during the second and third cold periods correspond to the relatively low variances of ENSO during the same periods; however, during the first cold period the temperatures correspond to a high ENSO variance. Moreover, the PDO index compared two lower values and a high value during the same periods. This shows that the temperature in the West Qinling Mountains was affected by multiple factors. The fourth-cold period, which is observed in the reconstruction for the middle of the Qilian Mountains, and the southeastern area of the Tibetan Plateau (Figure 6), may represent a larger scale cold event; precipitation during this time was low. The ENSO variance and the PDO index show two cold phases, with cold periods evident in the PDO and ENSO records during the 1950s and 1960s, as observed in Figure 6; a cold and wet period also occurs during this interval. However, in contrast to the explanation given for the above mentioned cold periods, this cold period may be explained by a stronger Indian summer monsoon [77]. Thus, local climate anomalies such as these serve to illustrate the importance of increasing our understanding of past climate variability, not only at large spatial scales, but also at local and regional levels.

## Conclusions

Tree-ring samples from six sites in the West Qinling Mountains of China were used to develop six new tree-ring width chronologies. In combination with 67 other proxy records of various types, the new chronologies were used to reconstruct temperatures in the West Qinling Mountains from AD 1500 to the present. Our results show that a hybrid-PPR method can effectively assimilate different types of proxy data with different temporal resolutions, and preserve more information about local climate variability than other similar techniques. The reconstruction shows 1) five distinct cold periods, at approximately AD 1520–1535, AD 1560–1575, AD 1610–1620, AD 1850–1875, and AD 1965–1985, and four warm periods, at approximately AD 1645–1660, AD 1705–1725, AD 1785–1795 and AD 1920–1945 during the past 500 years in the West Qinling Mountains; and 2) the 20^th^ century was not the warmest period during the past 500 years in the West Qinling Mountains; and 3) there is a dominant and persistent oscillation of ca.64 years during the period AD 1640–1790.
